# Effect of Gold Nanoparticles on the Physical Properties of an Epoxy Resin

**DOI:** 10.3390/ijms24065638

**Published:** 2023-03-15

**Authors:** F. Fraga-López, Lisbeth Jiménez Carrillo, María Pilar Vázquez-Tato, Julio A. Seijas, Francisco Meijide, José Vázquez Tato, Aida Jover

**Affiliations:** 1 Departamento de Física Aplicada, Facultad de Ciencias, Universidad de Santiago de Compostela, Avda. Alfonso X El Sabio s/n, 27002 Lugo, Spain; 2 Laboratorio de Investigación y Tecnología de Polímeros, Universidad Nacional de Costa Rica, Heredia 86-3000, Costa Rica; 3 Departamento de Química Orgánica, Facultad de Ciencias, Universidad de Santiago de Compostela, Avda. Alfonso X El Sabio s/n, 27002 Lugo, Spain; 4 Departamento de Química Física, Facultad de Ciencias, Universidad de Santiago de Compostela, Avda. Alfonso X El Sabio s/n, 27002 Lugo, Spain

**Keywords:** bisphenol A diglycidyl ether (DGEBA), *m*-xylylenediamine, gold nanoparticles, epoxy resin, glass transition temperature, degree of conversion

## Abstract

The effect of doping the bisphenol A diglycidyl ether (DGEBA)/m-xylylenediamine (mXDA) system with gold nanoparticles (AuNP) has been studied with differential scanning calorimetry (DSC), thermogravimetric analysis, dynamic mechanical analysis (DMA), and dielectric analysis (DEA). The evolved heat (Δ*H_t_*), the glass transition temperature (*T_g_*), and the associated activation energies of this relaxation process have been determined. Below a certain concentration of AuNPs (=8.5%, in mg AuNP/g epoxy matrix), *T_g_* decreases linearly with the concentration of AuNPs, but above it, *T_g_* is not affected. The degree of conversion *α* of this epoxy system was analyzed by the semiempirical Kamal’s model, evidencing that diffusion correction is required at high values of *α*. Activation energy values suggest that AuNPs can cause some impediments at the beginning of the crosslinking process (*n*-order mechanism). The slight difference between the initial decomposition temperature, as well as the temperature for which the degradation rate is at a maximum, for both systems can be accepted to be within experimental error. Mechanical properties (tension, compression, and bending tests) are not affected by the presence of AuNPs. Dielectric measurements show the existence of a second *T_g_* at high temperatures, which was analyzed using the Tsagarapoulos and Eisenberg model of the mobility restrictions of network chains bound to the filler.

## 1. Introduction

Natural Epoxy resins are widely used for many applications due to their versatility [1]. Frequently, additives (fillers) are incorporated into the resin to modify properties such as viscosity, shrinking, thermal expansion, and anticorrosion or to improve their mechanical properties [2]. This strategy is an alternative to the development of a new resin [3], which is always a more expensive process. The kinetic features of epoxy nanocomposites with fillers have been reviewed recently by Irzhak [4].

The incorporation of nanoparticles as fillers of epoxy resins is a research field attracting growing interest. The addition of fillers can modify the electrical performance of epoxy resins, and they are often used in high-voltage apparatuses as insulators. The literature reports evidence that contrary effects may be obtained. Thus, silver nanoparticles increase the conductance of an epoxy resin, having been observed that, qualitatively, the samples display most of the expected behavior predicted by percolation theories [5]. Similarly, when insulator nanoparticles are added to the resin, a decrease in that property may be observed. With respect to unfilled resin, in an epoxy-ZnO nanocomposite system, permittivity decreases with an increment of ZnO at low filler concentrations, but the opposite is observed above a threshold concentration [6]. Similar effects have been observed for SiO_2_ and Al_2_O_3_ fillers [7]. 

Akib et al. [8] performed the synthesis of metal nanoparticles (Au, Ag, and Cu) in an aqueous phase and their subsequent transfer to an epoxy resin. Close-packed particle arrangements (especially for gold nanoparticles, AuNP) and increments of the glass transition temperature of the epoxy resin due to the presence of nanoparticles were observed. Poly(methyl methacrylate)-coated AuNPs have been used for reinforcing hybrid membranes (prepared via the curing of bisphenol A diglycidyl ether (DGEBA) with poly(ethylene glycol)diamine) for the removal of heavy metal ions [9]. The dispersion of AuNPs into epoxy resins can create practical applications in the development of optical devices [10] and sensors.

Lednický and Bonyár [11] have prepared surface AuNP/epoxy nanocomposites for the successful label-free detection of DNA. AuNPs were used to decorate the surface of graphene/epoxy resin nanosheets to enhance the Raman sensitivity for detecting different concentrations of dopamine in human serum as a real sample [12]. Chen et al. [13] have used a direct synthesis of a AuNP monolayer on the surface of an epoxy resin SU-8 under UV exposure, providing a high specific surface area for applications of surface-enhanced Raman spectroscopy. The device was demonstrated to be useful in the analysis of malachite green (a commonly used biocide in aquaculture) under the limits of the European Union, as well as in the analysis of Rhodamine 6G (detection limit 10^−10^ M). The optical limiting threshold was also investigated in AuNP/epoxy resin systems [11]. Demir et al. [14] observed that the glass transition temperature of the DGEBA resin increases with the concentration of AuNPs, while thermal stability was slightly reduced and the effect on the mechanical properties was insignificant. Dong et al. [15] incorporated AuNPs into an epoxy matrix for photothermal curing, which enables the selective curing of an arbitrary shape within a liquid bath of epoxy, leaving the remainder unaltered but maintaining the mechanical properties of the cured epoxy. AuNPs were uniformly dispersed using poly(ethylene oxide) chains in an epoxy matrix based on DGEBA cured with a mixture of n-dodecylamine and m-xylylenediamine (mXDA). The composite exhibits photothermal effects, allowing for excellent shape memory properties [16]. A synergistic effect between multi-walled carbon nanotubes (MWCNT) and AuNPs and improved electromagnetic shield properties were observed for nanocomposites (epoxy-MWCNT-AuNP), with AuNPs being prepared by an impregnation method over organoclay [17]. Graphene/AuNP/epoxy composites were also prepared and used for the reduction of H_2_O_2_, with good linear response and high sensitivity, and for the catalytic reduction of 4-nitrophenol [18].

On the other hand, the microstructural perfection of the materials increases by minimizing the size of potential defects [2], with the size of the nano-filler being crucial for obtaining the good mechanical properties of brittle epoxy resins. Consequently, the experimental conditions for the synthesis and the dispersion control are critical parameters in the elaboration of these systems [3] when searching for the desired properties. AuNPs are commonly incorporated into the resin before starting the curing process, and only a few studies have been published in which the nanoparticles were obtained in situ within the resin [19].

The main aim of this paper was to perform a dielectric analysis of the DGEBA *n* = 0/m-XDA epoxy resin dopped with AuNPs that were incorporated in situ. This is a study to be conducted prior to any potential application of the new composite in electrical devices. However, as NPs could modify the curing process and the epoxy resin’s structure, the most important mechanical and thermodynamic properties must be determined. As the DGEBA/mXDA system has been an object of preferential attention during the last two decades, a comparison between both systems is feasible. For this last system, activation energies [20,21], optimum temperature range for the use of this material [22], transition glass temperature and the variation of the specific heat capacity [23], relaxation times [24], and the effect of calcium carbonate as filler [25] have been determined. This large background of data facilitates any comparative study. The partial characterization of the system in the presence of AuNPs, which is closely related to the present contribution, has also been published [26,27,28].

## 2. Results and Discussion

**Figure 1:**
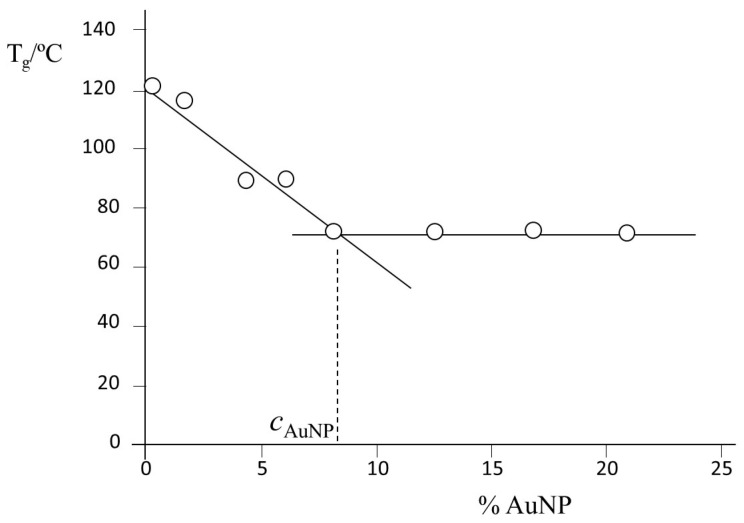
Dependence of the glass transition temperature (*T_g_*) of the DGEBA/mXDA/AuNP nanocomposite with the amount of AuNPs.

Calorimetric scans were performed by DSC, operating in a. dynamic mode between 40 and 140 °C at a heating ratio of 10 °C/min, to determine the glass transition temperature (*T_g_*) of resins prepared with different amounts of AuNPs. Figure 1 shows the dependence of *T_g_* with the amount of AuNPs (*T_g_*(°C) = (121.4 ± 4.1) − (6.08 ± 0.79) × [AuNP, %]; r^2^ = 0.9508); it remains constant above 8.5% AuNPs, the average value being 69.95 ± 0.14 °C. Most remaining experiments were carried out at this concentration of 8.5% AuNPs.

**Figure 2:**
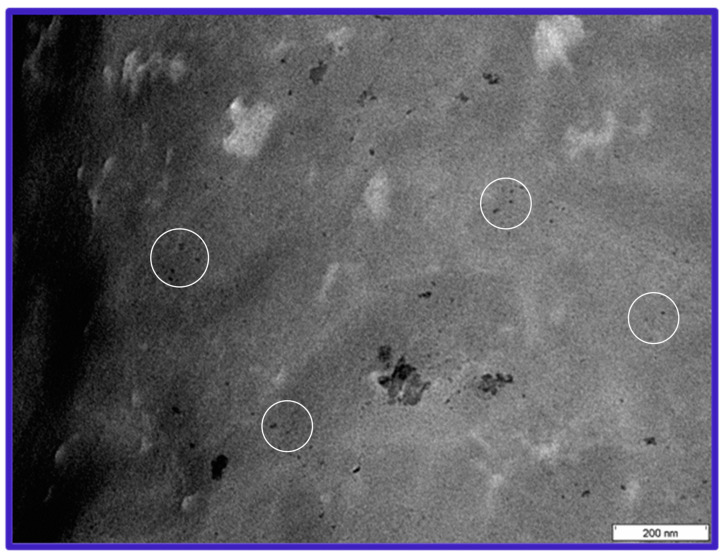
TEM image of the composite DGEBA (*n* = 0)/mXDA/AuNP (8.5%). White circles: Examples of isolated AuNPs.

Often, an increase in *T_g_* of epoxy resins has been observed when increasing the amount of added NPs [29,30,31]. It has been ascribed to the adsorption of polymers onto the particle’s surface, which reduces the polymer’s mobility and modifies the conformation of chain segments. Less common is the observation of a reduction in *T_g_*. This is the case of an epoxy resin with various amounts of carbon black or carbon nanofibers, silver nanoparticles [32], and epoxy/alumina nanocomposites [33]. Complex behavior has been observed with the addition of CNT on CNT composites [34]. Liu et al. [33] speculated that the reduction in *T_g_* is due to an uneven distribution of hardener molecules, thus leading to a stoichiometric imbalance between epoxy components. This is not the case for the present composite, as we have observed (Figure 2) that AuNPs are uniformly distributed in the epoxy resin. Dorigato et al. [32] have proposed that, simultaneously with the adsorption of polymers onto the particle surface, polymer–filler chemical interactions and the increment in viscosity reduce the crosslinking degree of the epoxy-net and drop *T_g_*.

However, according to Tsagaropoulos and Eisenberg [35], the addition of nanoparticles leads to the formation of tightly bound and loosely bound polymer chains around the filler particles. At very low filler concentrations, with the average interparticle distance being large, the percentage of the resin network close to the nanoparticles will also be low, and most of the resin will remain in the absence of the filler. Consequently, the *T_g_* value will be close to the unmodified resin. However, at large filler concentrations, the average interparticle distance is low, most of the resin network is tightly bound to nanoparticles, and most of the resin is in a continuous phase of restricted mobility, which will exhibit its own Tg value. The incorporation of more filler particles decreases the average interparticle distance, and loosely bound regions are gradually transformed into tightly bound ones. In other words, the mobility restrictions become progressively severe. At an intermediate concentration of the filler, the average interparticle distance will correspond to a “critical” value. The behavior shown in Figure 1 is consistent with this qualitative analysis, and above 8.5%, the further addition of AuNPs will not modify the status of the resin as the full network is already tightly bound to nanoparticles.

Transmission electron microscopy (TEM) images show a fine distribution of the AuNPs in the epoxy composite (Figure 2), the size of the individual particles being less than 50 nm. They are mostly spherical in shape, which is expected from the reduction process followed [36]. Some agglomerates are also present, possibly formed during the preparation of the nanocomposite as a consequence of the attraction of the nanoparticles due to their high surface energy, as they do not have any surface treatment for helping the dispersion maintenance.

**Figure 3:**
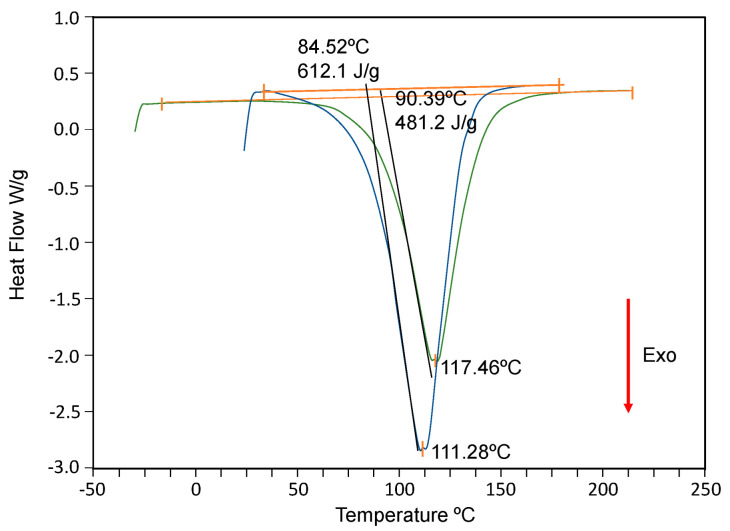
DSC scans of the DGEBA/mXDA (blue line, 111.28 °C [27]) and DGEBA/mXDA/AuNP systems (117,46 °C. black line).

The dynamic regime (10 °C/min from −30 °C to 250 °C) was used to determine the total enthalpy of curing (Δ*H_T_*), the obtained value being Δ*H_o_* = −483 ± 5 J g^−1^ (Figure 3). This value is significantly lower than those reported by Paz et al. (=−555 J/g) [37] and Fraga et al. [27] (=−612 ± 6 J g^−1^) for the DGEBA/m-XDA system (without AuNPs). The curing reaction of the system with AuNPs is less exothermic, suggesting that nanoparticles can cause steric hindrances that reduce crosslinking, in agreement with the observed drop in *T_g_*.

### 2.1. Kinetic Analysis

Calorimetric tests were carried out in the isothermal mode from 40 to 100 ° C in intervals of 10 °C, allowing for the measurement of partial enthalpies and the determination of the degree of conversion *α* of this epoxy system at each temperature, according to Equation (1):

(1) $$\alpha =\frac{\Delta H_{t}\;}{\Delta H_{o}}$$

where Δ*H_t_* is the partial area under a DSC curve up to time *t* at the curing temperature *T*, and Δ*H_o_* is the total heat evolved during the curing reaction. The results are shown in Table 1.

**Figure 4:**
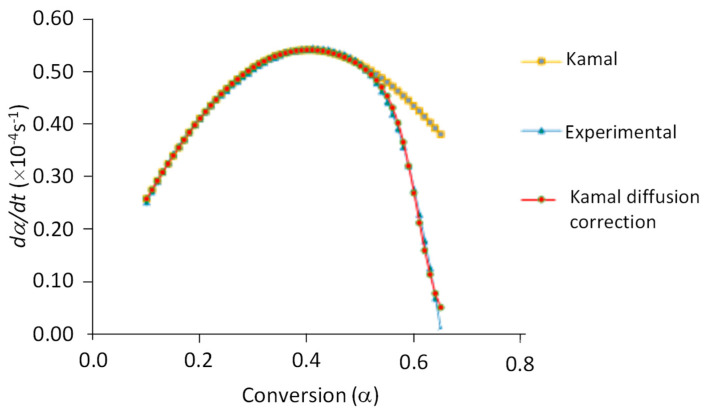
Experimental conversion rate (*dα*/*dt*) vs. conversion (*α*) at 40 °C of the curing reaction of the composite DGEBA/mXDA/AuNP, 8.5%. Fittings according to Kamal’s model (Equation (2)), with and without diffusion corrections, are also shown.

It can be observed that *α* increases with *T* until 80 °C, and it remains constant above it, within experimental error. The maximum conversion observed (*α* = 0.95) is in agreement with the observations made by Paz et al. [37] for this system without AuNPs. Simultaneously, the reaction rates, *dα*/*dt*, were measured. Figure 4 shows an example of the dependence of *dα*/*dt* with *α* at 40 °C. Similar behavior is observed at other curing temperatures.

Previous results were analyzed according to the semiempirical Kamal’s model (Equation (2)) with and without diffusion correction.

(2) $$\left(\frac{d\alpha }{dt}\right)=(k_{1}+k_{2}\alpha^{m}){\left(1-\alpha \right)}^{n}F\left(\alpha \right)\;$$

In this equation, *k*_1_ and *k*_2_ are kinetic constants, and *m* and *n* are the reaction orders corresponding to the autocatalytic and *n*-th order mechanisms, respectively. *F*(*α*) is equal to 1 when the diffusion correction is not required; otherwise, it is given by Equation (3) [38].

(3) $$F\left(\alpha \right)={\left[1+e^{A_{1}\left(\alpha -\alpha_{c}\right)}\right]}^{-1}$$

Here, *A*_1_ (diffusion coefficient) and *α_c_* (critical conversion) are empirical parameters that depend on the curing temperature. For low values of *α*, the effect of diffusion is negligible, F(α)≈1, and the reaction is kinetically controlled. When *α* approaches *α_c_* and beyond that point, *F*(*α*) decreases, and the reaction becomes diffusion-controlled. 

All previous parameters were determined by the non-linear fitting of experimental data. The obtained values are shown in Table 2.

It can be noticed that the experimental data conform to the proposed model. The critical conversion values are always above 60%, and they are comparable with those observed for the system without AuNPs for which values between 0.716 (50 °C) and 0.978 (110 °C) have been published [39]. A value of *α_c_* close to one means that the diffusion control only becomes important at the end of the crosslinking process.

The global order of reaction *m + n* ≅ 3 is constant in the range of curing temperatures studied. For this system without AuNPs, Paz et al. [37] obtained a reasonable correlation for a global order of 3, but the best fitting corresponds to a global order of 2.5 (*m =* 1 and *n* = 1.5). 

From the variation of *k*_1_ and *k*_2_ with temperature activation energies and pre-exponential factors (Arrhenius equation) were obtained. The values are shown in Table 3. For both mechanisms, the activation energies are much lower than those corresponding to the system without AuNPs. They are, however, within the range of published values for resins obtained with DGEBA and other secondary amines [40,41,42]. The comparison of values in Table 3 suggests that the AuNPs hinder the beginning of crosslinking in the *n*-order mechanism, requiring higher activation energy. 

### 2.2. Thermogravimetric Analysis

**Figure 5:**
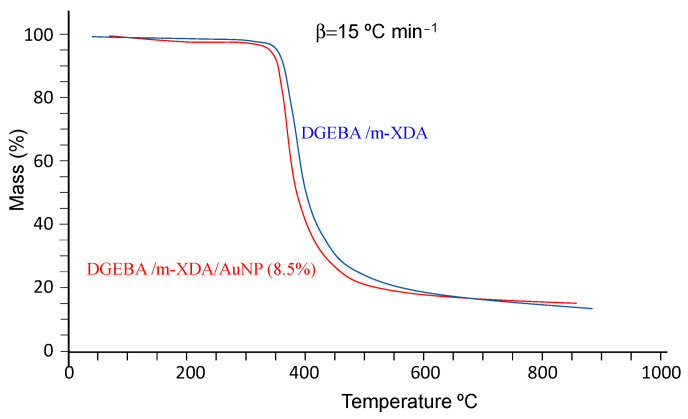
Thermogravimetry experiments for the DGEBA/m-XDA/AuNP (8.5%) at different heating rates (β).

Figure 5 shows the mass loss for the DGEBA/mXDA/AuNP (8.5%) system as a function of temperature at different heating rates (β). The mass loss curves correspond to a single decomposition profile with well-defined initial and final temperatures, being of the C type [43]. This behavior is characteristic of epoxy materials in a nitrogen atmosphere [20,44]. In a solid state, these decomposition reactions are slow, being controlled by the rate of heat transfer from the reaction interface. 

At different heating rates, the initial decomposition temperatures (*T_i_*, determined at the 5% mass loss point) [45] are between 329 °C and 364 °C (Table 4), suggesting a high crosslinking density. This is expected behavior for thermoset materials [20,44,46]. Table 4 also shows the residue after the decomposition process (determined at 850 °C) and the temperature values of the inflection point of the mass loss curves, *T_m_*, at which the degradation rate is at a maximum. Such values are slightly higher than those of the pristine system [47]. However, by taking into account the common error in these determinations, which is around 3–5 K, the difference can be accepted to be within experimental error.

The values of *T_m_* and *β* may be used to estimate the activation energy of the thermodegradation reaction. According to Kissinger [48] the slope of the plot of $ln\left(\beta /T_{m}^{2}\right)\;\mathrm{vs}.\;T_{m}^{-1}$ provides the value of the activation energy, *E_a_*. From the values of Table 4, a value of *E_a_* = 169 kJ mol^−1^ (r^2^ = 0.9683) is obtained. This value is considerably lower than those of 222 kJ mol^−1^ (r^2^ = 0.9759) and 210 kJ mol^−1^ (r^2^ = 0.9841) obtained by [47] and [20], respectively, for the system without AuNPs.

Alternatively, other methods may be used for the estimation of the activation energy (see ref. [20] for a quick presentation). Among them, the Flynn–Wall–Ozawa method [49,50] is widely used, as it does not require the knowledge of the reaction order (as Kissinger’s does). Ozawa [51] has indicated that this method is more precise and simpler than Kissinger’s method. The method uses Doyle’s approximation [52], and consequently, it is limited to conversions in the interval of 11–26%. Table 5 shows the obtained values together with those for the DGEBA/mXDA system [20,47]. Clearly, *E_a_* varies with *α.* Consequently, according to Vyazoykin and Sbirrazzuoli [53], as the method assumes that the activation energy is constant, systematic errors in *E_a_* may be introduced. Even so, all *E_a_* values for DGEBA/mXDA/AuNP (8.5%) are lower than for DGEBA/m-XDA. These results are in contrast with observations for other fillers and resins [54,55]. 

### 2.3. Compression and Flexural Tests

**Figure 6:**
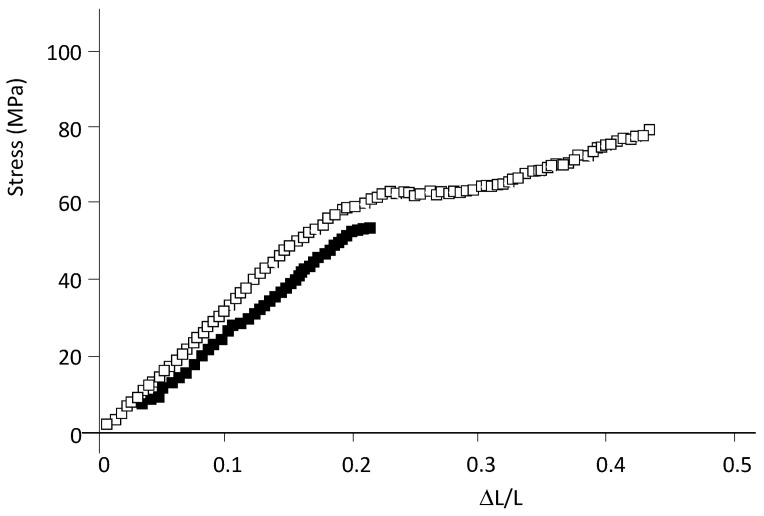
Stress–strain curves from compression tests for DGEBA (*n* = 0)/mXDA /AuNP (8.5%) (□) and DGEBA (*n* = 0)/mXDA (■).

Figure 6 shows examples of stress vs. strain curves obtained from compression tests for DGEBA (*n* = 0)/mXDA/AuNP (8.5%) and DGEBA (*n* = 0)/m-XDA systems. Curves are typical of compression tests. At low deformations, an elastic region (linear dependence) is observed, followed by the plastic region and the densification regime. Table 6 shows the Young’s modulus determined from the linear region of the stress–strain curves for both systems. The proximity of both values, together with their standard deviation, suggests that the gold nanoparticles do not alter the compression properties. 

**Figure 7:**
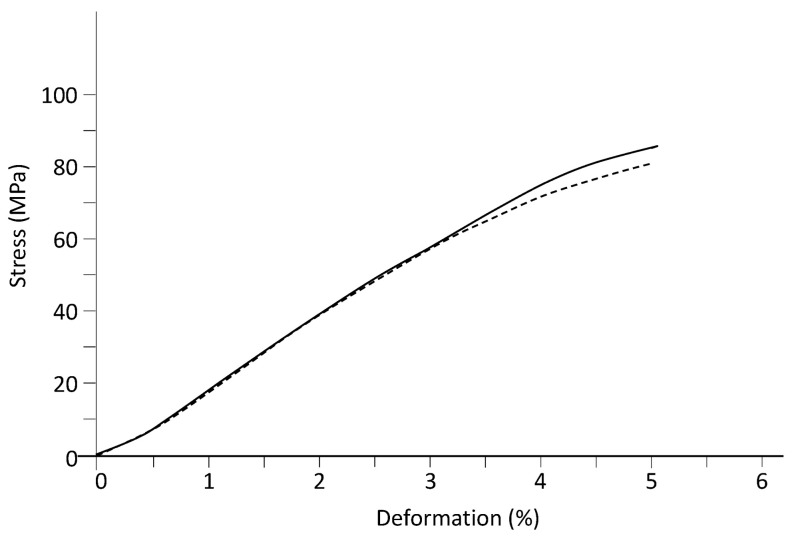
Stress–strain curves (average values) from the three-point bending flexural test for the DGEBA/mXDA system: without AuNPs (dashed line) and with AuNPs (8.5%) (solid line).

The modulus of elasticity in bending was determined by the three-point bending flexural test for both systems. Figure 7 shows typical experimental curves. From the curves, the stress at 5% strain and the bending modulus were determined (Table 6). When the experimental error is considered, the obtained values for both parameters of the two systems were not statistically different, suggesting that AuNPs do not alter bending properties and cannot be considered true reinforcement particles.

Other studies involving nanoparticles report improvements in mechanical properties with the addition of small amounts of NPs. According to Zheng et al. [56], nanoparticles tend to occupy holes in the epoxy resin, resulting in a reduction in the total free volume and an increase in the cross-linking density. In the present case, DSC experiments suggest a small reduction in the cross-linking density in the presence of AuNPs, and consequently, a reinforcement of mechanical properties was not observed.

### 2.4. Dynamic Mechanical Analysis

**Figure 8:**
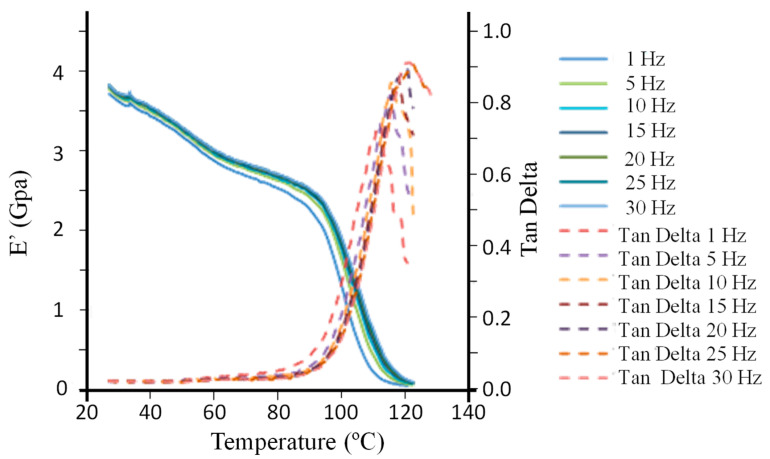
Temperature dependence of the storage modulus, *E*′, and *tan δ* at frequencies between 1 and 30 Hz for the DGEBA/mXDA/AuNP (8.5%).

Figure 8 shows the behavior of the storage modulus, *E’*, and *tan δ* of the DGEBA (*n* = 0)/mXDA/AuNP (8.5%) nanocomposite at various frequency values. 

At low temperatures, the storage modulus decreases slightly. This is related to a reduction in the stiffness of the material caused by γ transitions (increasing the free volume caused by movements of bonds) and β transitions (movements inside chains and adjacent atoms in the main chain) [57]. Around 90 °C, there is an abrupt drop in the storage modulus, reflecting a sudden change in the elastic behavior of the material. This is related to the beginning of a coordinated movement of large sections of chains in amorphous regions [57]. The storage modulus curves exhibit an inflection point above 100 °C, which may be used for the measurement of glass transition temperature *T_g_*. However, *T_g_* is also measured from the peaks of the loss modulus and *tan δ*, this final one occurring at a higher temperature than the *T_g_* measured via the storage modulus onset, which in turn is higher than the one measured from the storage modulus. Any of these *T_g_* values can increase with the oscillation frequency, as the glass transition is a kinetic transition [58]. Because of this frequency dependence, a frequency of 1 Hz has customarily been used as a standard value [58]. On the other hand, such dependence has been used to determine the activation energy, *E_a_*, by applying an Arrhenius-like equation (Equation (4)):

(4) $$f=f_{o}e^{-\left(E_{a}/RT_{g}\right)}$$

with *f_o_* being a pre-exponential factor. For its application, the *T_g_* values determined from *tan δ* peaks, at frequencies ≤ 10 Hz, have been used, according to conclusions from Li et al. [59]. These criteria have been used to recalculate *E_a_* from measurements published for DGEBA/mXDA [47,60,61]. Table 7 shows the obtained results for the resin DGEBA/mXDA with AuNPs (8.5%) and without AuNPs (recalculated from published results).

It must be remarked that the *E_a_* values have been obtained by using only three frequencies (1, 5, and 10 Hz). Measurements provided by L. Fraga [47] show a rather high dispersity evidenced by the low value for the correlation coefficient *r*^2^ and the high standard deviation of the activation energy (equal to 22%). Student’s t-tests indicate that the difference between the *E_a_* value obtained for DGEBA/mXDA/AuNP (8.5%) is not statistically different from the *E_a_* values obtained for DGEBA/mXDA by Fraga et al. [60] and Núñez et al. [61]. 

### 2.5. Dielectric Analysis

**Figure 9:**
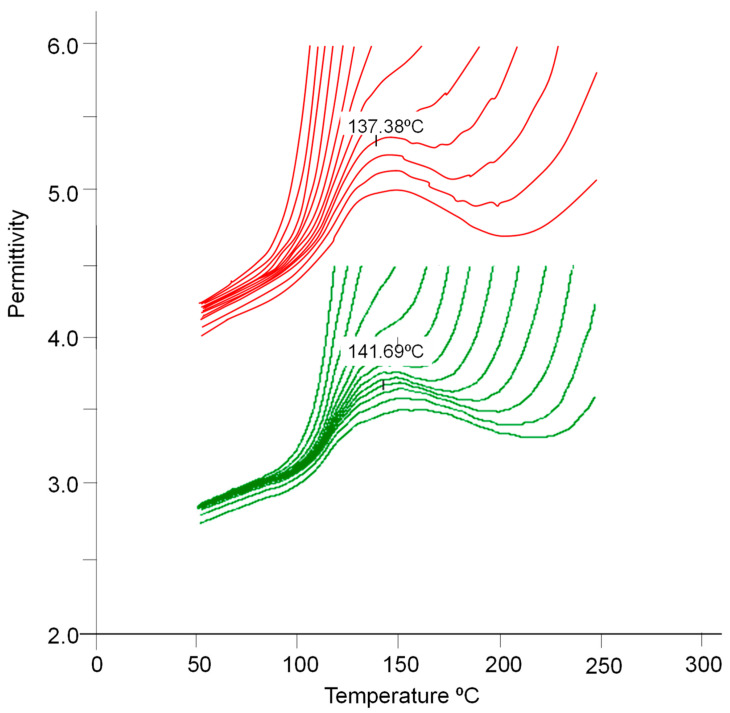
Permittivity *ε’* vs. temperature at various applied frequencies for the system DGEBA/mXDA without (green lines) and with AuNPs (8.5%) (red lines).

**Figure 10:**
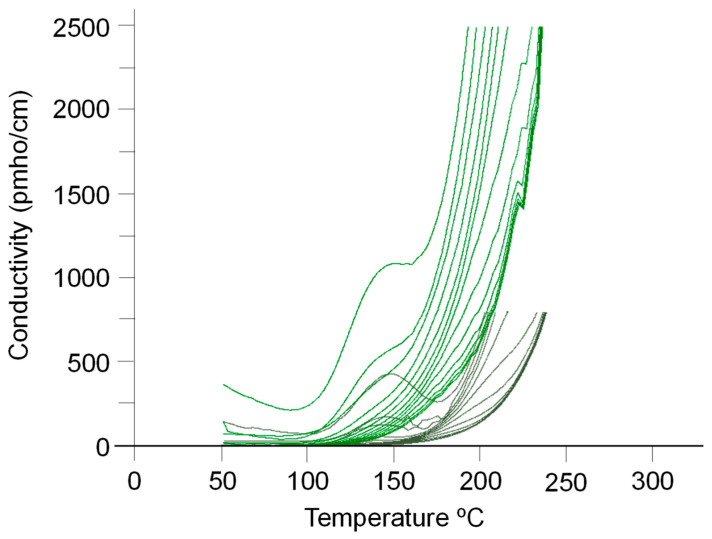
Conductivity *σ* as a function of the temperature at different applied frequencies for the system DGEBA/mXDA without (grey color) and with AuNPs (8.5%) (green color).

**Figure 11:**
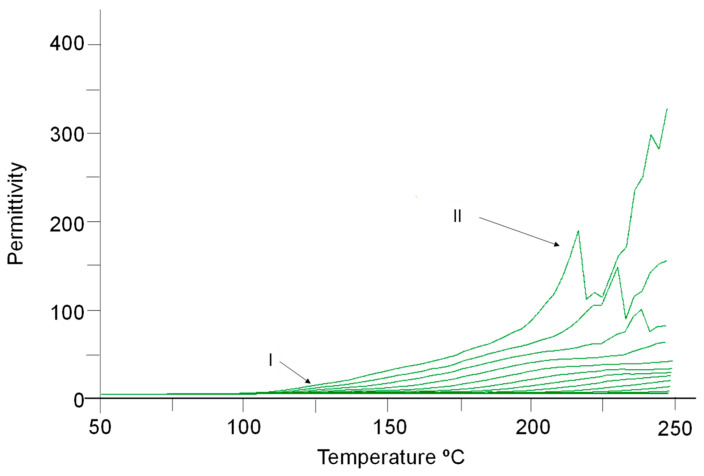
Permittivity *ε*′ vs. temperature at various applied frequencies for the system DGEBA/mXDA/AuNP (8.5%). Arrows indicate the existence of two transitions: I (see Figure 9) and II, Tsagaropoulos and Eisenberg transition.

We have carried out a dielectric analysis of the DGEBA/m-XDA resin without and with AuNPs (8.5%). This analysis provides the permittivity (*ε*′) and loss factor (*ε*″). From these two quantities, dissipation (*tan δ* = *ε*″/*ε*′) and conductivity were obtained (*σ* = 2π *ε_o_ε*″*f*; *f*, applied frequency; *ε_o_*, the absolute permittivity in free space). Their dependence on the temperature at the applied frequencies was also obtained. Results are shown in Figure 9, Figure 10 and Figure 11.

Figure 9 shows the typical behavior of permittivity for a crosslinked amorphous network, evidencing the appearance of *α* transitions or relaxations (maximum of each curve). The temperature value of the maximum of the permittivity curves is associated with the *T_g_* value of the material. Table 8 shows *T_g_* values for the two systems at 464 Hz. As observed by other techniques, the addition of AuNPs reduces the glass transition temperature. As the frequency increases, such transitions shift towards higher temperature values. *T_g_* is also observed by *tan δ*. 

It is known that the effective permittivity of epoxy nanocomposites is governed by the polarization of the epoxy, the nanoparticles, and the interfacial polarization at the interface between them. The permittivity of epoxy resin is related to the number of orientable dipoles and their facility for orientation under the applied electric field. It may be observed that the conductivity decreases as frequency increases. Such dependence has already been commented on by other authors such as Wang and Chen for the LY556 epoxy resin dopped with SiO_2_ or Al_2_O_3_ particles [7].

Figure 9 and Table 8 evidence that the addition of AuNPs (8.5%) to DGEBA/mXDA enlarges the permittivity of the system, the increment being almost 1.5 times at 1 kHz. Analogously, the loss factor (Table 8, 4th column) increases 2.5 times with the addition of AuNPs. We must recall that the 8.5% concentration of AuNPs corresponds to a critical value, above which nanoparticles do not have a strong influence on crosslinking, as DSC experiments suggest. At this concentration, a large percentage of nanoparticles (as for other critical definitions, such as the *critical micelle concentration* of surfactants) will probably be present as free (unbound) fillers in the nanocomposite. If this is the case, the nanocomposite DGEBA/mXDA/AuNP (8.5%) will have a much higher conductance than the pristine system. Figure 10 and Table 8 show that this is the case. The influence of fillers on permittivity is complex and depends on the type of filler, size, concentration, and temperature. At concentrations of less than 1% (a threshold concentration at which permittivity shows a minimum value), the permittivity for both epoxy-SiO_2_ (or Al_2_O_3_) nanocomposites decreases; however, above the threshold value, the permittivity of nanocomposites begins to increase with the filler’s concentration [7]. Similar behavior has been observed for nanosized TiO_2_ and ZnO fillers. However, these last two fillers increase the permittivity at all frequencies when they are micron-sized [6]. At 50 Hz and 200 °C, TiO_2_ increases the permittivity more than three times, but the effect is null at 20 °C [35]. At 100 °C, the permittivity of the nanocomposite increases with the amount of added MWCNT [62]. 

A closer inspection of the permittivity vs. the temperature for the dopped system, at different frequencies, evidences the existence of two characteristic peaks (Figure 11). The first one, known as the *α* transition, is associated with the orientation of the functional groups of the side chains in the structure of the material (Figure 9). The second peak may be related to the mobility restrictions imposed by the filler (see above), a concept introduced by Tsagaropoulos and Eisenberg [35]. This second glass transition would correspond to regions containing those chains of reduced mobility and will appear only after an overlap of the regions of the loosely bound network is achieved. This can occur only at high temperatures above 220 °C.

## 3. Materials and Methods

A differential scanning calorimeter from TA Instruments (New Castle, DE, USA; DSC Q20 model, equipped with a refrigerated cooling system, USA) was used for calorimetric studies. The evolved heat (Δ*H_t_*) was obtained at a temperature range from −30 °C to 250 °C with a heating rate of 10 °C min^−1^. The glass transition temperature (*T_g_*) was determined in a second scan at an identical range of temperature and heating rate. The isothermal mode was used for measuring partial enthalpies at curing temperatures of 70–100 °C. In all these experiments, samples weighing between 9 and 13 mg were poured into aluminum pans. Experiments were carried out under a dry nitrogen atmosphere. The nanocomposites were cured as previously described by Fraga et al. [27,28]. The material was pre-cured at 40 °C for 24 h, and the final cured was at 130 °C for 30 min.

Thermogravimetric analysis was carried out in a TA Instruments (New Castle, DE, USA; TGA Q500 model). The analyses were performed at the dynamic mode between 25 °C and 900 °C, at rates between 5 and 30 °C/min (at intervals of 5 °C) under a dry nitrogen atmosphere. The sample’s mass was 7 or 8 mg. An average of five experiments were conducted at each experimental condition for DSC and thermogravimetric measurements.

DMA experiments were performed in a PerkinElmer (Waltham, MA, USA; DMA 8000 model). Dynamic mechanical thermal studies were carried out on samples heated from room temperature to 150 °C at 2 °C/min, at frequencies between 1 and 30 Hz, and at intervals of 5 Hz. Typical dimensions of cured prismatic shape specimens were 20 × 9 × 1 mm.

Compression tests were performed in a Metrotec Hounsfield H-10 KM, T02000 (MI, USA) with an available range of 0.5–500 mm/min. The samples were prepared in a waxed cylindrical steel mold. Samples were cured at 40 °C for 24 h and post-cured at 130 °C for 30 min. After the curing time, samples (typical dimension being diameter 7 mm and height 9 mm) were compressed at five rates between 5 and 70 mm/min. Stress was within the interval of 0–80 MPa until the samples broke. Experiments were repeated three times.

Bending tests were carried out in an INSTRON 3365 instrument (Norwood, MA; USA). Essays can be performed for values < 5 kN, 1000 mm/min and vertical space length of 1193 mm (maximum values). For measuring the bending elasticity modulus, the specimen (rectangular shape obtained from cured composites) rested on two supports that were 31 mm apart. Load was applied at its center at 1.3 mm/min to reach 5% bending or until it broke. Average values from six measurements, were obtained. 

A TA Instruments (New Castle, DE, USA; DEA 2970 model) was used for dielectric measurements. The typical dimensions of the prismatic samples were 2.5 × 2.5 × 0.06 mm. The frequency of 1 kHz was used as a reference since it was the one used for temperature calibration. The value obtained at this frequency for the calibration of polycarbonate film (standard) was 168 °C, which is very close to that reported in the literature (165 °C). Dielectric experiments were performed at temperatures within the range of 35–260°C at a heating rate of 1.4°C/min. The applied force was 300 N, and the distance between plates was 0.5 mm, similarly to the thickness of the sample. 

TEM images were obtained at room temperature in a JEOL JEM-1011 (Tokio, Japan), which was operated at 80 kV and equipped with a MegaView III camera. For the analysis, samples were cut as thin membranes (<70 nm) in an ultramicrotome and deposited onto carbon-coated copper grids at room temperature. 

### Sample Preparation

The epoxy resin DGEBA was Resin 332 from Sigma Chemical Co (St. Louis, MO, USA), having an equivalent molecular mass of 173.6 g mol^–1^; the cross-linking agent was m-xylylenediamine (mXDA) (Sigma); the gold salt (HAuCl_4_) was from Sigma Chemical Co; the sodium borohydride was from Panreac Chemical (Barcelona, Spain).

The reduction process (Au^3+^ → Au^0^) was carried out in situ, within the resin, to favor their dispersion and avoid the agglomeration processes of the nanoparticles. Methanol was chosen as the solvent since it solubilizes both the gold salt (HAuCl_4_) and the epoxy resin DGEBA. Later, methanol evaporated at 70 °C. 

In a typical preparation, HAuCl_4_ in methanol (0.8 mL, 5 mM) was added to DGEBA (7 g), also in methanol, at 45 °C. The mixture was shaken at 65–70 °C for 24 h. A methanol solution of sodium borohydride (1.2 mL) was added, and a gradual color change (from yellow to dark pink) was observed. The reduction was completed after 24 h. The mixture was kept at 65–70 °C with constant stirring to remove the methanol. Then, mXDA (1.4 mL) was added, keeping the stoichiometric ratio with the resin. The solution was poured into square or cylindrical metal molds according to the requirements of the tests to be carried out. The amount of AuNPs is expressed as the total Au concentration and is given as (mg AuNP/g epoxy matrix).

## 4. Conclusions

Although the amount of added AuNPs to the epoxy resin DGEBA/mXDA is low (8.5% in mg AuNPs/g epoxy matrix), the permittivity of the system enlarged 1.5 times at 1 kHz. Similarly, the loss factor and conductance increased 2.5 times with respect to the pristine system. The dielectric measurements show the existence of a second *T_g_*. It has been analyzed according to the Tsagarapoulos and Eisenberg model of the mobility restrictions of network chains bound to the filler. However, the addition of AuNPs does not modify the maximum conversion of the system or its mechanical properties. The initial stages of the curing reaction are controlled by chemical kinetics; however, above a critical degree of conversion, the reaction becomes diffusion-controlled.

## Figures and Tables

**Table 1:** Enthalpy of the reaction, Δ*H*(*T*), obtained by the isothermal mode and conversion degree, *α*, at different curing temperatures.

Temperature (°C)	−Δ*H* (J/g)	*α*
40	334 ± 67	0.69 ± 0.14
50	360 ± 61	0.75 ± 0.12
60	412 ± 49	0.85 ± 0.10
70	422 ± 48	0.88 ± 0.10
80	458 ± 24	0.95 ± 0.06
90	452 ± 25	0.94 ± 0.06
100	451 ± 25	0.93 ± 0.06

**Table 2:** Kinetic constants, reaction orders for the autocatalytic and *n*-th order mechanisms, and values for *A*_1_ and *α_c_*, according to Equation (3), at different curing temperatures.

Parameter	Temperature (°C)				
	40	50	60	70	80
*k*_1_/(10^−4^ s^−1^)	0.094 ± 0.02	0.50 ± 0.03	1.90 ± 0.01	3.10 ± 0.02	8.90 ± 0.04
*k*_2_/(10^−4^ s^−1^)	3.80 ± 0.10	9.10 ± 0.13	18.7 ± 0.09	30.4 ± 0.07	63.2 ± 0.23
*m*	1.25	1.20	1.50	1.40	1.53
*n*	1.72	1.78	1.51	1.59	1.49
*m + n*	2.97	2.98	3.01	2.99	3.02
*A*_1_	47.34	42.24	48.95	59.77	83.00
*α_c_*	0.610	0.649	0.782	0.825	0.926

**Table 3:** Activation energies and pre-exponential factors (Arrhenius equation) for the *n*-th order and autocatalytic mechanisms.

System		*n* Order Mechanism	Autocatalytic Mechanism
DGEBA/mXDA/AuNP (8.5%)	*E_a_* (kJ mol^−1^)	100 ± 3	63 ± 4
	*A*(s^−1^)	8.40 ± 0.03 (×10^11^)	1.24 ± 0.11 (×10^7^)
DGEBA/mXDA [37]	*E_a_* (kJ mol^−1^)	61.03	45.14
	*A* (s^−1^)	7.17 × 10^7^	1.68 × 10^6^

**Table 4:** Degradation parameters at different heating rates of the system DGEBA/mXDA/AuNP (8.5%) nanocomposite.

Heating Rate (*β*) °C/min	DGEBA/m-XDA/AuNP (8.5%)			DGEBA/m-XDA [47]
	*T_i_* °C (5% Decomposition)	% Residue (at 850 °C)	T_m_ °C	T_m_ °C
5	329.00	18.16	357.9	353.8
10	346.17	17.86	366.0	365.0
15	349.07	14.88	374.6	365.0
20	361.34	14.25	380.9	372.3
25	359.46	14.76	389.1	376.3
30	364.39	11.97	389.4	380.0

**Table 5:** Activation energies obtained by the Flynn–Wall–Ozawa method for DGEBA/m-XDA/AuNP (8.5%) and DGEBA/m-XDA [20,47] systems.

	DGEBA/m-XDA/AuNP (8.5%)		DGEBA/m-XDA [47]	DGEBA/m-XDA [20]	
*α* (%) *	*E_a_* (kJ/mol)	R^2^	*E_a_* (kJ/mol)	*α* (%)	*E_a_* (kJ/mol)
10	151 ± 3	0.9897	202.7	5	148.6
13	156 ± 2	0.9970	219.3	8	157.0
16	160 ± 1	0.9992	229.2	11	188.4
19	160 ± 1	0.9990	235.1	14	208.8
22	163 ± 2	0.9989	238.7	17	220.9
25	163 ± 1	0.9985	241.2	20	229.2

* *α* values for the DGEBA/m-XDA system were within 1% higher than the ones indicated here

**Table 6:** Young’s modulus and bending modules for DGEBA/mXDA/AuNP (8.5%) and DGEBA (*n* = 0)/mXDA systems.

Property	Young’s Modulus (MPa)	Maximum Elasticity Limit (MPa)	Compressive Strength (MPa)	Stress at 5% Strain (MPa)	Bending Modulus (MPa)
DGEBA (*n* = 0)/m-XDA/AuNP (8.5%)	320 ± 4	60.4± 3.6	86 ± 13	87 ± 6	(2.22 ± 0.19) × 10^3^
DGEBA (*n* = 0)/m-XDA	307 ± 27	58.7 ± 2.9	85 ± 11	76 ± 5	(2.01 ± 0.18) × 10^3^

**Table 7:** Activation energy from *T_g_* values determined from *tan δ* peaks at frequencies ≤ 10 Hz and *T_g_* values measured at 1 Hz.

	DGEBA/m-XDA			DGEBA/m-XDA/AuNP (8.5%)
References	[47]	[60]	[61]	This paper
*E_a_* (kJ/mol^−1^)	461 ± 100 (*r*^2^ = 0.9549)	583 ± 44 (*r*^2^ = 0.9943)	640 ± 41 (*r*^2^ = 0.9959)	597 ± 68 (*r*^2^ = 0.9871)
*T_g_* (°C) at 1 Hz	124.0	118.4	118.9	111.0

**Table 8:** Glass transition temperature *T_g_*, permittivity *ε*′, loss factor *ε*″, dissipation *tan δ*, and conductivity (pmho/cm) for the system DGEBA/mXDA without and with AuNPs (8.5%). All of them are at 1 kHz, except *T_g_.*

HAuCl_4_ (%)	*T_g_* (°C) at 464 Hz	*ε*′	*ε*″	*tan δ*	*σ* (pmho/cm)
0.0	141.7	3.68	0.1062	0.02928	432.9
8.5	137.4	5.34	0.3375	0.06080	1087

## Data Availability

Not applicable.
